# Quantification of Proteus syndrome-associated lung disease

**DOI:** 10.1186/s13023-023-03013-9

**Published:** 2024-02-06

**Authors:** Christopher A. Ours, Anna Buser, Mia B. Hodges, Marcus Y. Chen, Julie C. Sapp, Bernadette R. Gochuico, Leslie G. Biesecker

**Affiliations:** 1 Center for Precision Health Research, National Human Genome Research Institute, National Institutes of Health, Bethesda, MD 20892 USA; 2 Section of Inflammation and Cardiovascular Diseases, National Heart, Lung, and Blood Institute, National Institutes of Health, Bethesda, MD USA; 3 Medical Genetics Branch, National Human Genome Research Institute, National Institutes of Health, Bethesda, MD USA

**Keywords:** Proteus syndrome, Lung disease, Outcome assessment

## Abstract

**Background:**

Proteus syndrome is an ultra-rare mosaic overgrowth disorder. Individuals with Proteus syndrome can develop emphysematous and cystic changes of the lung that may lead to progressive respiratory symptoms and require surgical intervention. This retrospective study seeks to quantify the radiographic features of Proteus syndrome-associated lung disease using computed tomography (CT) of the chest. The first method derives a Cystic Lung Score (CLS) by using a computer-aided diagnostic tool to quantify the fraction of cystic involvement of the lung. The second method yields a Clinician Visual Score (CVS), an observer reported scale of severity based on multiple radiographic features. The aim of this study was to determine if these measurements are associated with clinical symptoms, pulmonary function test (PFT) measurements, and if they may be used to assess progression of pulmonary disease.

**Results:**

One hundred and thirteen imaging studies from 44 individuals with Proteus syndrome were included. Dyspnea and oxygen use were each associated with higher CLS (*p* = 0.001 and < 0.001, respectively) and higher CVS (*p* < 0.001 and < 0.001). Decreases in percent predicted FVC, FEV_1_, and DLCO each correlated with increased CLS and CVS. The annual increase of CLS in children, 5.6, was significantly greater than in adults, 1.6. (*p* = 0.03). The annual increase in CVS in children, 0.4, was similar to adults, 0.2 (*p* = 0.36).

**Conclusions:**

Proteus syndrome-associated lung disease is progressive. The rate of cystic progression is increased in children. Increased scores in CLS and CVS were associated with clinical symptoms and decreased pulmonary function. Both methods were able to detect change over time and were associated with clinically meaningful outcomes which may enable their use in interventional studies.

## Background

**Fig. 1:**
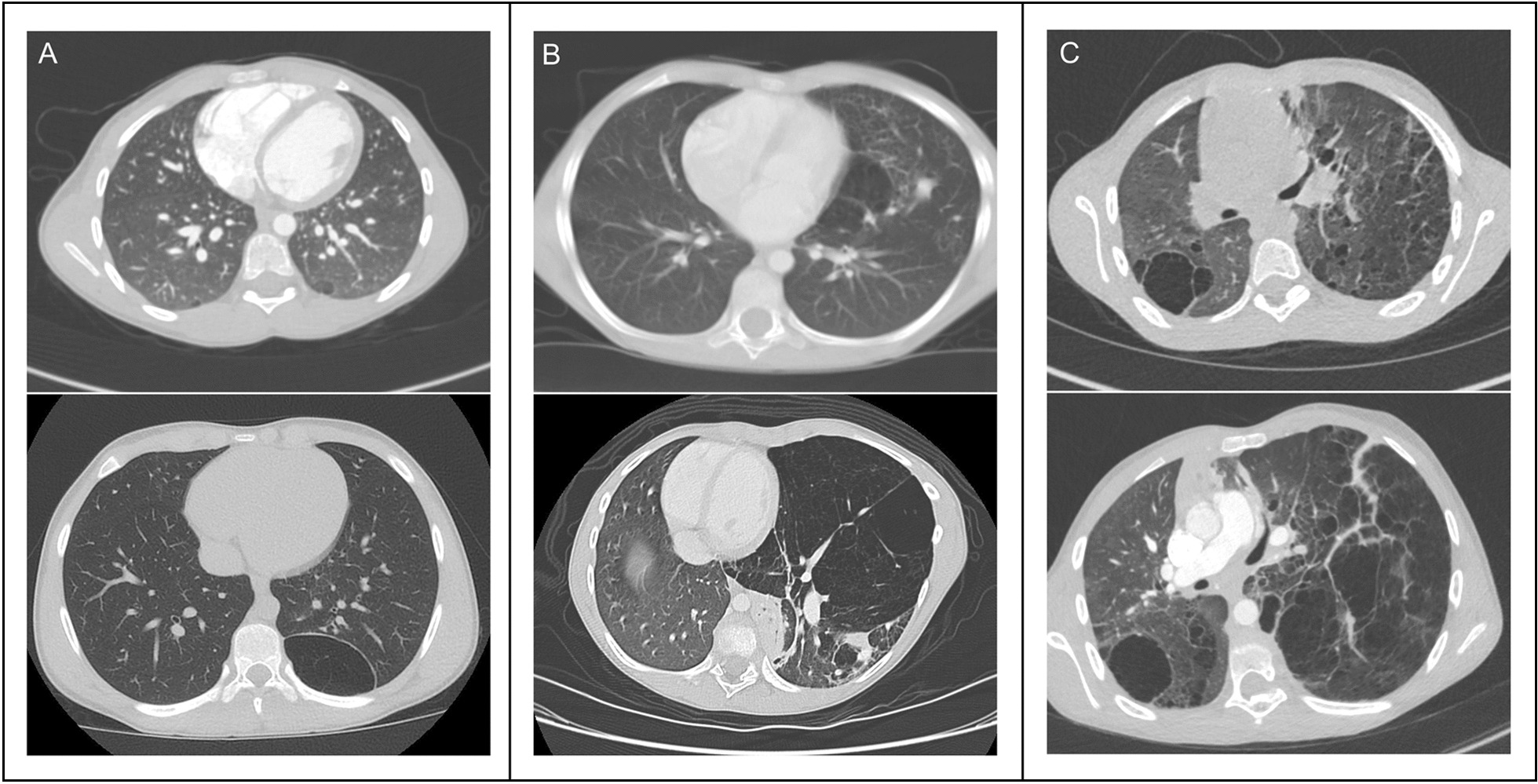
Radiographic progression of lung disease in three individuals **A**, **B**, **C** at seven years old (top) and after three or four years (bottom)

Proteus syndrome is a sporadic and progressive segmental overgrowth disorder, which can affect skin, bone, subcutaneous tissue, and other organs. It is caused by a mosaic activating *AKT1* variant, most often c.49G > A, p.(Glu17Lys) [10]. Pulmonary involvement of Proteus syndrome results in emphysematous and cystic lung disease often accompanied by scoliosis and chest wall deformity [4, 5, 8, 9, 11, 14, 16, 17, 25]. The spectrum of this pulmonary disease ranges in severity from clinically silent small cysts to progressive diffuse involvement and chronic respiratory failure [4, 25]. The radiographic features, most easily discerned by computed tomography (CT), include hyperlucency of the lung parenchyma, ground glass opacifications, distal pulmonary vein dilation, and nodules [13] (Fig. 1). A systematic literature review suggests that the prevalence of bullous lung disease in Proteus syndrome is 9.3% although more recent data suggests a prevalence of approximately 36% [6, 24]. Resection of affected lung segments has been therapeutic for symptomatic individuals with regionally limited disease [8, 25]. However, individuals with diffuse involvement may require lung transplantation [16].

Although previous reports have documented the severity of emphysematous and cystic changes, no study has quantified the progression of Proteus syndrome-associated lung disease. A quantitative approach to Proteus syndrome-associated lung disease can be used to further describe the condition and be used to evaluate the efficacy of investigational interventions. In this study, we sought two methods of quantifying the severity of the Proteus syndrome associated lung disease using CT imaging of the chest. The first is a computer-aided diagnostic system to quantify cystic areas using low attenuation values. Density-volumetric analyses of CT scans have previously been used to quantify the fraction of lung compromised by cysts in lymphangioleiomyomatosis, chronic obstructive pulmonary disease, and reported in an individual with Proteus syndrome [3, 4, 11]. The second is a point system we developed that relies on clinician interpretation of the severity of three radiographic features: cysts, hyperlucent lung parenchyma, and ground glass. Similar methods using advanced imaging have been proposed in multiple pulmonary diseases including bronchopulmonary dysplasia, cystic fibrosis, and emphysema [1, 2, 21]. An imaging-based scoring system for cystic fibrosis have been used as endpoints in clinical trials [18, 23].

These methods may enhance our understanding of the natural history of Proteus syndrome and play a role in the assessment of therapeutic effect in future clinical trials. We present these two methods to measure of lung disease in Proteus syndrome, their association with clinical features, measurements over time, and discuss the advantages and disadvantages of each.

## Methods

### Participant selection

Individuals enrolled in the Natural History of Proteus Syndrome study (94-HG-0132) from January 2000 to November 2022 were reviewed. Those with a clinical or clinical-molecular diagnosis of Proteus syndrome based on previously established diagnostic criteria who had at least one CT imaging study of the chest were included. The diagnostic criteria comprise a point system based on clinical features to diagnose Proteus syndrome and helps distinguish it from other overgrowth disorders [19]. Only imaging studies prior to lung surgery or resection were included. Individuals suspected of having alternative etiologies of cystic lung disease such as environmental and smoking related emphysema, systemic sclerosis, amyloidosis, lymphocytic interstitial pneumonia, or lymphangioleiomyomatosis (LAM) based on imaging characteristics and clinical history were excluded. This study was approved by the institutional review board of the National Institutes of Health and all participants, or their legal guardians provided written consent. The CT imaging studies were sourced from the film library of the National Institutes of Health Clinical Center (NIHCC) and from multiple other institutions. Some of the radiographs and data in this study overlap with recent publications from the Natural History of Proteus Syndrome study [6, 13].

### Cystic Lung Score

**Fig. 2:**
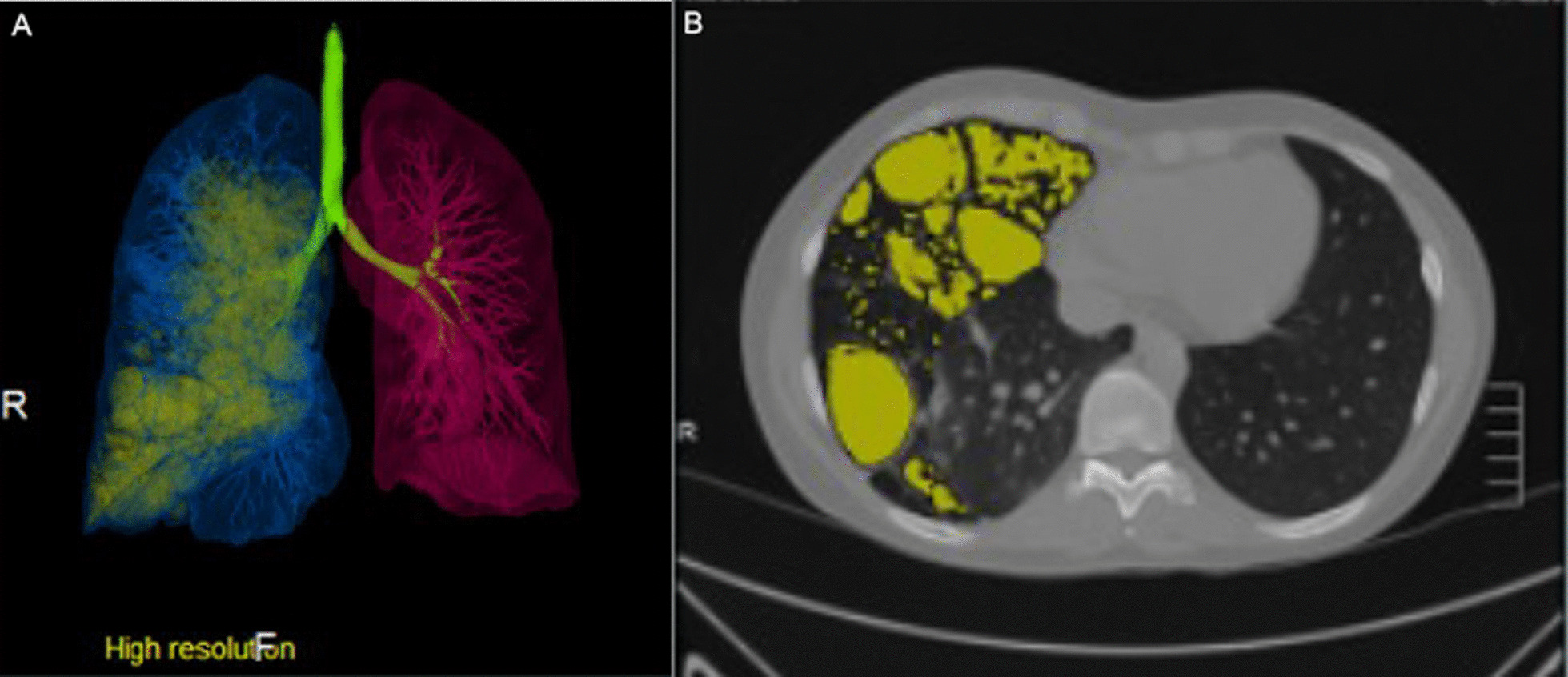
**A** Computer-assisted lung volume analysis showing reconstructed volumes of right lung (blue), left lung (red), and cystic spaces (yellow). **B** Axial image of same imaging study highlighting cystic spaces (yellow)

The software used to quantify pulmonary cysts was Lung Volume Analysis (Canon Medical Systems, Otawara-shi, Japan). This software has been used to measure cysts in lymphangioleiomyomatosis [3]. This method relies on region-growing algorithms to determine lung volume beginning with seed points in the right and left lung and a histogram-based technique to exclude vascular spaces and bronchi. The cystic regions were defined by identifying the volume of low attenuation using an adaptive Hounsfield unit threshold (Fig. 2). The total volume of the cystic regions was divided by the total lung volume to yield a Cystic Lung Score (CLS). A threshold CLS of five was used to include an individual in the change over time analysis.

### Clinician Visual Score

The non-cystic radiographic features of lung disease in Proteus syndrome such as hyperlucent lung parenchyma and ground glass are not captured using the computer-assisted density-volumetric approach. Therefore, we developed a point system to include these features. Computed tomography images were reviewed independently by two physicians (CAO and BRG) for the presence of cysts, ground glass, and hyperlucent lung parenchyma. The presence of cysts, ground glass, and hyperlucent lung parenchyma were graded based on reviewer estimated fraction of total lung involvement (Absent (0%), Minimal (< 5%), Mild (5–25%), Moderate (25–50%), and Severe (> 50%)). Each grade of cysts, ground glass and hyperlucent lung parenchyma was assigned a severity-based numeric value (Absent = 0, Minimal = 1, Mild = 2, Moderate = 3, Severe = 4). The overall Clinician Visual Score (CVS) was derived by the sum of severity-based values of each feature yielding a score on a scale from 0 to 12. A threshold CLS of four was used to include an individual in the change over time analysis.

### Clinical status and pulmonary function tests

Participant demographics, clinical status, and pulmonary function tests (PFT) were obtained at the NIHCC as part of the ongoing natural history study. Clinical status was ascertained by review of the NIHCC clinical record for the presence of dyspnea on exertion and for chronic supplemental oxygen requirement. Clinical data concurrent with the most recent imaging study were not always available as some were sourced from other institutions. If clinical data concurrent with the most recent imaging study were not available, then prior clinical records from the NIHCC were used to determine clinical status. Pulmonary function tests were included only if the interpretation indicated adequate effort and reproducibility and if obtained within one month of a corresponding CT imaging study. There were a limited number of repeated measures for PFTs thus the results for individuals with two PFTs were averaged in the analysis yielding set of PFT measures per individual when available.

### Statistical analyses

The general characteristics of the study population are presented using descriptive statistics. Intraclass correlation coefficients (ICC (2,1)) and unweighted Cohen’s Kappa were calculated to assess reliability of the CVS and its components between the two raters. The comparisons of CLS and CVS measurements to dyspnea and oxygen requirement by the Wilcoxon rank sum test were made using only the most recent imaging study. Correlations of PFT measures with density-volume or point-based measurements were performed using Pearson’s method.

Progression of lung cysts was evaluated using a mixed model with a random slope by individual to account for repeated measures. Given the previously observed age-dependent differences in Proteus syndrome progression of the cerebriform connective tissue nevus the model included an interaction term to assess the difference in adults (≥ 18 years of age) and children (< 18 years of age) [15]. All models and statistical calculations were performed in R Version 4.1.2 using the RStudio Version 2021.09.1.

## Results

### Study population

Eighty-two participants in the Natural History of Proteus Syndrome study (94-HG-0132) were reviewed for the availability of CT chest imaging. A total of 123 imaging studies from 44 individuals were identified. Of these, 10 imaging studies were excluded because they were obtained after a lung surgery. Serial images were available from 26 individuals. The cohort included 15 females and 29 males. No individuals reported a history of smoking. Forty-three individuals had a confirmed mosaic *AKT1* variant. We were unable to confirm the *AKT1* status of the remaining individual because an affected tissue sample was not available. Their presentation with cerebriform connective tissue nevus, bony overgrowth, central nervous system asymmetry, epidermal nevi and a vascular malformation is sufficient for a clinical diagnosis of Proteus syndrome [19]. Six individuals had surgical treatment of their lung disease. The clinical indications for the earliest scan were: rule out pulmonary embolism (18), evaluate for lung disease (9), abnormal PFT (6), unknown (5), pneumonia (2), metastatic evaluation (2), adenopathy (1), scoliosis (1) (Table 1).

**Table 1:** Participant characteristics

	N = 44
Age at earliest CT scan, mean (SD)	14.9 (11.6) years
*Sex*	
Male	29 (65%)
Female	15 (35%)
*Diagnosis*	
Clinical-molecular proteus syndrome	43 (98%)
Clinical Proteus syndrome	1 (2%)
Surgical treatment	6 (14%)
*Indication for earliest CT scan*	
Rule out pulmonary embolism	18 (41%)
Evaluate for lung disease	9 (20%)
Abnormal PFT	6 (14%)
Unknown	5 (11%)
Pneumonia	2 (5%)
Metastatic evaluation	2 (5%)
Adenopathy	1 (2%)
Scoliosis	1 (2%)

### Cystic Lung Score

**Fig. 3:**
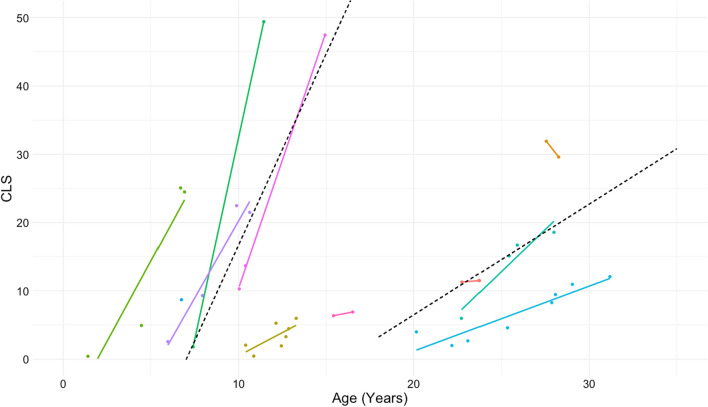
Change in CLS in the 11 individuals with CLS score > 5 on at least one imaging study. Each color represents repeated measurements per individual. The dashed lines show the modeled 5.6 point annual increase for children and 1.6 point annual increase for adults

Eleven (25%) individuals had a CLS of five or greater on at least one imaging study. The annual increase in CLS during childhood (age < 18 years) was 5.6 and in adulthood was 1.6. The Cystic Lung Score increased more rapidly in children compared to adults (*p* = 0.03) (Fig. 3).

### Clinician Visual Score

After resolution of two raters, minimal or greater cystic features were found in 26 (59%), ground glass in 28 (64%), and hyperlucent lung parenchyma 26 (59%) of individuals on the most recent imaging study. The entire range of the CVS (0 to 12) was represented in the cohort with a plurality of individuals, 12 (27%), having a score of zero. There were 21 (48%) individuals who had a CVS of four or greater on at least one imaging study. The frequency of CVS and the frequency of the components of the CVS from participants’ most recent imaging study are in Table 2.

**Table 2:** Components and total CVS of most recent CT imaging study

Components and total Clinician Visual Score			
Components of Clinician Visual Score	Frequency	Clinician Visual Score	Frequency
Cysts			
Yes	26 (59%)	0	12 (27%)
Minimal	8	1	3 (7%)
Mild	11	2	7 (16%)
Moderate	3	3	2 (5%)
Severe	4	4	1 (2%)
No	18 (41%)	5	3 (7%)
		6	5 (11%)
Ground glass		7	2 (5%)
Yes	28 (64%)	8	3 (7%)
Minimal	16	9	1 (2%)
Mild	7	10	2 (5%)
Moderate	2	11	2 (5%)
Severe	3	12	1 (2%)
No	16 (36%)		
Hyperlucent Lung			
Yes	26 (59%)		
Minimal	6		
Mild	3		
Moderate	9		
Severe	8		
No	18 (41%)		

**Fig. 4:**
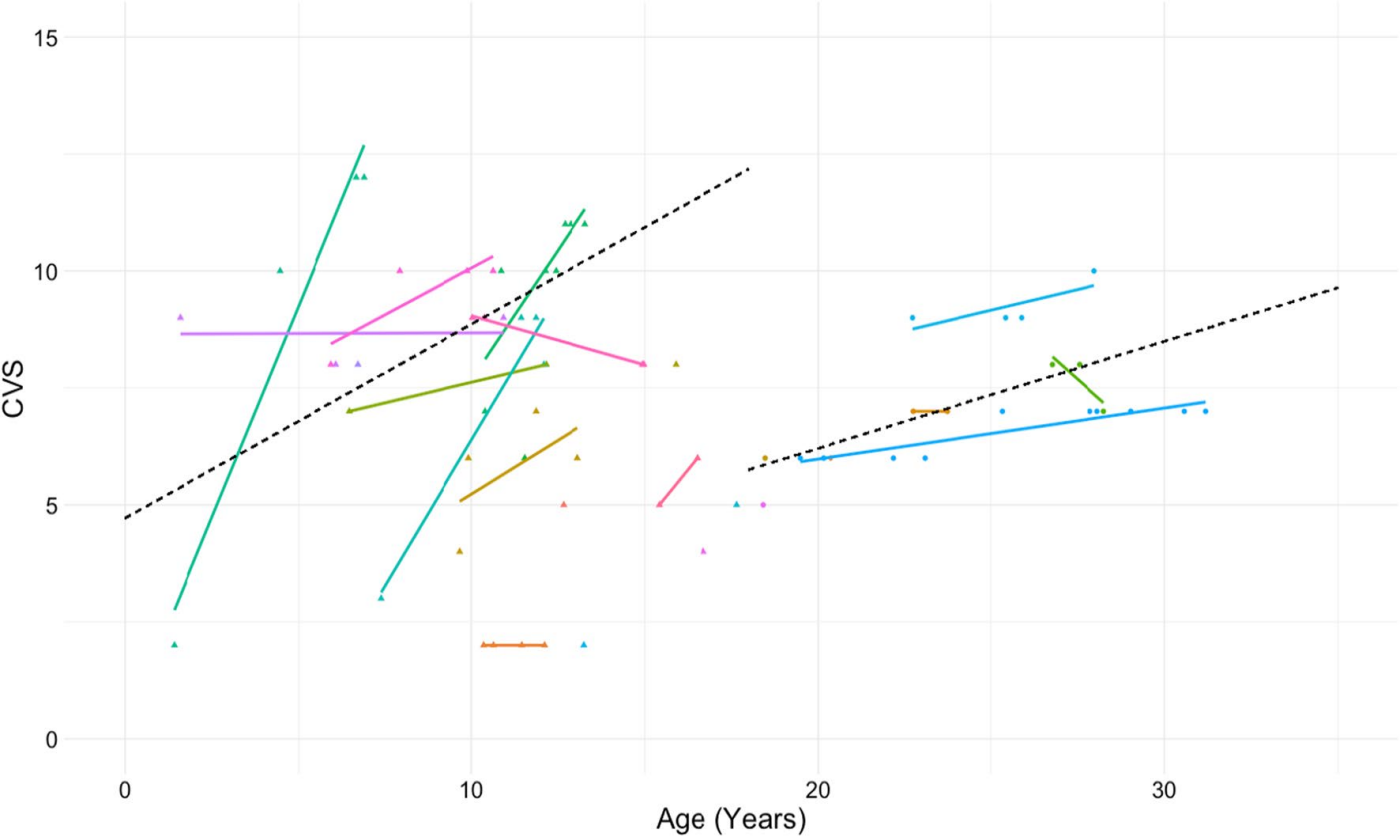
Change in CVS in the 21 individuals with CVS score > 3 on at least one imaging study. Each color represents repeated measurements per individual. The dashed lines show the modeled 0.4 annual increase for children and 0.2 annual increase for adults

The annual increase in CVS for children was 0.4 and in adults was 0.2. There was not a statistically significant difference in the annual rate of CVS increase among adults and children (p = 0.36) (Fig. 4). The ICC of the total CVS between the two raters was 0.98 and unweighted Cohen’s Kappa was 0.66. This indicates a substantial agreement and suggests absolute disagreements were likely small in magnitude. The ICC and Kappa respectively for each component of the CVS was 0.96 and 0.66 for cysts, 0.93 and 0.8 for ground glass, and 0.93 and 0.8 for hyperlucent lung.

### Correlation of CLS and CVS with clinical status and pulmonary function tests

**Fig. 5:**
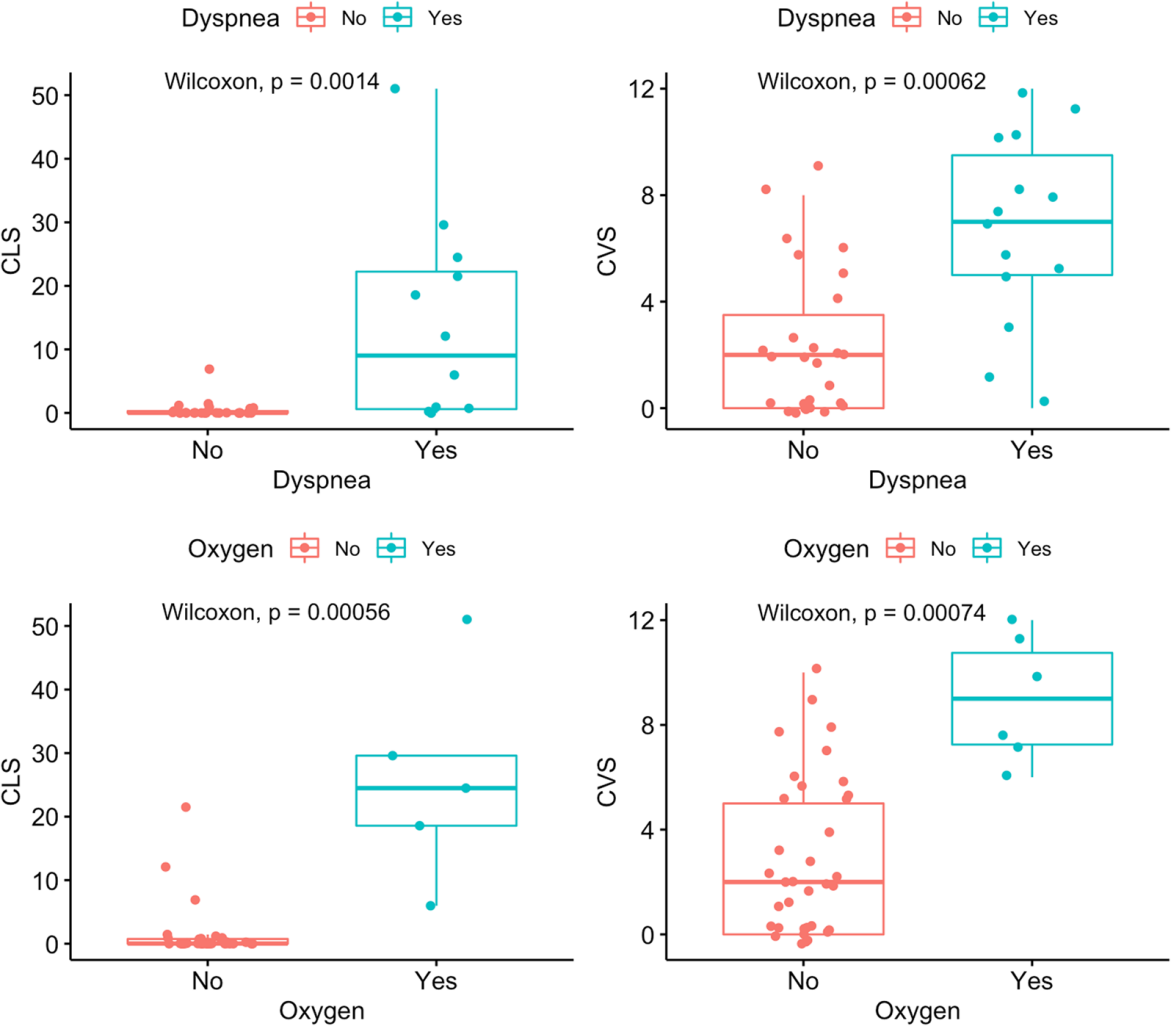
Dyspnea on exertion was present in 17 (39%) of individuals and was associated with increased CLS and CVS. Supplemental oxygen requirement was present in 7 (16%) of individuals and was also associated with increased CLS and CVS

**Fig. 6:**
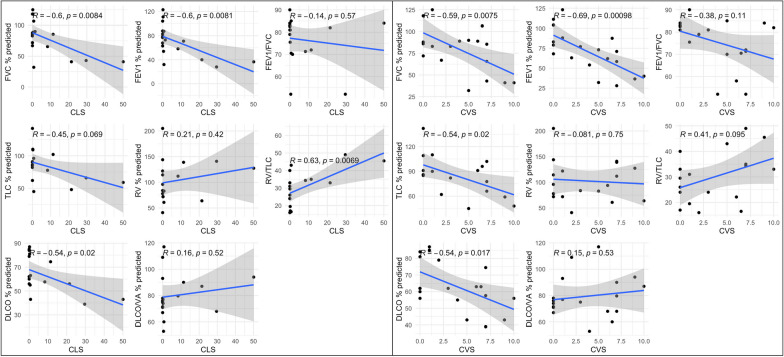
Correlation of CLS and CVS with PFT measurements. Each graph includes Pearson correlation coefficient and p-value

Dyspnea on exertion was reported by 17 (39%) individuals. Supplemental oxygen was prescribed to seven (16%) individuals. Dyspnea was associated with a higher CVS (*p* < 0.001) and higher CLS (*p* = 0.001). Supplemental oxygen use was also associated with a higher CVS (*p* < 0.001) and higher CLS (*p* < 0.001) (Fig. 5). There were 19 individuals with at least one PFT concurrent with a CT imaging study. Thirteen individuals had only one PFT and the remaining six had two PFTs. Multiple PFT measures were correlated with the CLS and CVS. The correlation of RV/TLC, percent predicted FVC, FEV_1_, and DLCO were statistically significant for CLS (*p* = 0.006, 0.008, 0.008, and 0.02, respectively) (Fig. 6). The correlation percent predicted FVC, FEV_1_, TLC, and DLCO were significant for CVS (*p* = 0.007, < 0.001, 0.02, 0.017, respectively). Pearson correlation coefficients and p-values are summarized in Table 3.

**Table 3:** Correlation of PFT measures with CLS and CVS

	CLS		CVS	
	Pearson coefficient	*p*-value	Pearson coefficient	*p*-value
FVC % predicted	− 0.6	0.0084*	− 0.59	0.0075*
FEV_1_% predicted	− 0.6	0.0081*	− 0.69	0.00098*
FEV_1_/FVC	− 0.14	0.57	− 0.38	0.11
TLC % predicted	− 0.45	0.069	− 0.54	0.02*
RV % predicted	0.21	0.42	− 0.081	0.75
RV/TLC	0.63	0.0069*	0.41	0.095
DLCO % predicted	− 0.54	0.02*	− 0.54	0.017*
DLCO/VA % predicted	0.16	0.52	0.15	0.53

*p-value < 0.05

## Discussion

Proteus syndrome-associated lung disease is a significant contributor to morbidity and mortality. While in some it may be clinically silent, progression may lead to dyspnea, chronic respiratory failure, or death. The onset of respiratory symptoms may occur in childhood or young adulthood. There are no proven pharmacologic therapies though given the obstructive pattern on PFTs some are treated with bronchodilators or inhaled corticosteroids. Surgical removal of the affected lung has been shown to improve PFTs but may have limited utility for those with diffuse disease. Radiographically, the lungs can have areas of hyperlucency, emphysematous changes, ground glass opacity, and diffuse enlarging cysts. This diverse set of findings and the presence of extrapulmonary manifestations of Proteus syndrome can distinguish it from other primary diffuse cystic lung diseases [6].

The differential of diffuse cystic lung disease includes a range of disorders including alpha-1-antitrypsin deficiency, lymphocytic interstitial pneumonia, Birt-Hogg-Dubé (BHD) syndrome, pulmonary Langerhans cell histiocytosis, and LAM. Extrapulmonary disease is an important consideration in the diagnosis. All participants in this study with lung disease had other features of Proteus syndrome, often overgrowth of bone or skin manifestations. Renal cysts and asymmetry may be seen in Proteus syndrome, however solid or vascular lesions would suggest a possible alternative diagnosis such as angiomyolipoma in LAM or renal cancer in BHD syndrome. Histologically, areas of proliferative myofibroblast are common in Proteus syndrome-associated lung disease while LAM and Langerhans cells are not present. Inflammation may be focal but is not a prominent feature. Radiographically, Proteus syndrome-associated lung disease is often associated with other pulmonary findings such as enlargement of pulmonary veins and mosaic distribution of hyperlucent lung and ground glass opacities and is not associated with lung regions such as the basal predominance in BHD [13].

The ability to quantify disease burden can improve our understanding of the natural history of a disorder and be useful in measuring the outcomes of a clinical intervention or therapeutic trial. The software methods to derive the CLS in this study has been used in LAM to quantify cystic burden, measure disease progression, treatment response, and predict the need for surgical treatment such as transplantation [22]. When applied to this study there were some significant differences. Participants in LAM studies were prone to minimize compression of the lungs by the heart and were mostly adults whereas this study used clinically obtained imaging studies in which individuals were supine and often children. The reliance on a Hounsfield unit threshold that captures the predominantly cystic disease of LAM may underestimate the radiographic extent of disease in Proteus syndrome due to frequent areas of hyperlucent lung or ground glass found in over half of individuals in this study. It is also important to recognize that in clinical trials, forced expiratory volume in one second (FEV_1_) rather than radiographic disease has been an accepted surrogate endpoint for efficacy. This includes pivotal studies that led to the regulatory approval of sirolimus for LAM [12].

We have applied two methods for measuring radiographic Proteus syndrome-associated lung disease and shown them to correlate with clinically important outcomes and demonstrate progressive disease over time. Nevertheless, each method has its own advantages and disadvantages.

The density-volumetric approach of the CLS has the advantage of reduced interpreter bias through use of an algorithm to quantify total lung and cystic volumes in contrast to the CVS which relies on clinician observer judgment. The CLS also provides a continuous scaled measurement with greater resolution compared to the CVS which includes integers 0 to 12. The resolution of the CLS increases sensitivity to year-to-year differences particularly in pediatric ages whereas the CVS also has a ceiling effect when either a single radiographic feature dominates or when multiple features are competing for involvement and radiographic severity. However, the CLS does not consider opacifying lesions such as ground glass or hyperlucent lung that does not meet the Hounsfield unit threshold and volume required to be considered cystic. An example of this is seen in the discordance of CLS and CVS in one individual whose CLS was below 10 over multiple imaging studies and CVS was 10 or greater in the same imaging studies. The CVS may be a more reflective measure of an individual with a predominately emphysematous or ground glass presentation.

This study had several limitations. First, Proteus syndrome-associated lung disease is present in only a fraction of this ultra-rare disease, thus, our cohort size is small. The size of the cohort and distribution of data mean relying on non-parametric analyses or relying on the ability of linear mixed-effects models to absorb violation of normality assumptions [20]. In addition, the statistical difference in the CLS progression in children versus adults must be interpreted with caution. Upon visual inspection, this difference may be driven by the youngest children who progress rapidly whereas adolescents may not exhibit as rapid of a progression. While the data do show an association with CLS and CVS with clinical symptoms, report of dyspnea extracted from clinical documentation is less reliable than systematic prospective assessment with a patient reported outcome measure. In addition, our data are too sparse to investigate thresholds of CLS or CVS that predict these clinical symptoms or prognosticate on need for surgical intervention.

This retrospective study is susceptible to ascertainment bias, favoring individuals with lung disease because advanced chest imaging is not the standard of care for screening individuals with Proteus syndrome for pulmonary involvement. To explore the magnitude of this bias, we sought the indication of each individual’s earliest scan and found a plurality (41%) were to rule out pulmonary embolism. This is not surprising given that Proteus syndrome is a known risk factor for venous thromboembolism [7]. Many of these studies were obtained at the NIHCC in response to an elevated D-dimer but not necessarily respiratory symptoms. The next most common indications were evaluation for lung disease and abnormal PFTs which may indicate these individuals had symptoms or other reason for the ordering clinician to suspect lung pathology. For these reasons, the presented frequency of lung disease may be an overestimate of the true prevalence of Proteus syndrome-associated lung disease. Another limitation is that the included imaging studies were heterogeneous in technique including use of intravenous contrast. We included scans not only obtained at the NIHCC but those performed at outside institutions. This also meant that nine imaging studies could not be measured for CLS due to the technique or due to metal artifact such as from spinal fusion.

## Conclusions

This study advances the quantification of Proteus syndrome disease burden through the application of two methods to measure radiographic lung disease. Both methods measured radiographic progression. The CLS showed the rate of cystic progression is increased in children. Increased CLS and CVS were associated with the presence of clinical symptoms and decreased pulmonary function. These findings support the use of these methods in future studies.

## Data Availability

Data sets generated during and/or analyzed during the present study are not publicly available due to participant privacy but are available from the corresponding author on reasonable request.
